# Identification of crucial aberrantly methylated and differentially expressed genes related to cervical cancer using an integrated bioinformatics analysis

**DOI:** 10.1042/BSR20194365

**Published:** 2020-05-12

**Authors:** Xiaoling Ma, Jinhui Liu, Hui Wang, Yi Jiang, Yicong Wan, Yankai Xia, Wenjun Cheng

**Affiliations:** 1 Department of Gynecology, The First Affiliated Hospital of Nanjing Medical University, Nanjing, China; 2 State Key Laboratory of Reproductive Medicine, Center for Global Health, School of Public Health, Nanjing Medical University, Nanjing, China; 3 State Key Laboratory of Modern Toxicology of Ministry of Education, School of Public Health, Nanjing Medical University, Nanjing, China

**Keywords:** Bioinformatics analysis, Cervical cancer, Differentially expressed genes, Gene Expression Omnibus (GEO), Methylation

## Abstract

Methylation functions in the pathogenesis of cervical cancer. In the present study, we applied an integrated bioinformatics analysis to identify the aberrantly methylated and differentially expressed genes (DEGS), and their related pathways in cervical cancer. Data of gene expression microarrays (GSE9750) and gene methylation microarrays (GSE46306) were gained from Gene Expression Omnibus (GEO) databases. Hub genes were identified by ‘limma’ packages and Venn diagram tool. Functional analysis was conducted by FunRich. Search Tool for the Retrieval of Interacting Genes Database (STRING) was used to analyze protein–protein interaction (PPI) information. Gene Expression Profiling Interactive Analysis (GEPIA), immunohistochemistry staining, and ROC curve analysis were conducted for validation. Gene Set Enrichment Analysis (GSEA) was also performed to identify potential functions.We retrieved two upregulated-hypomethylated oncogenes and eight downregulated-hypermethylated tumor suppressor genes (TSGs) for functional analysis. Hypomethylated and highly expressed genes (Hypo-HGs) were significantly enriched in cell cycle and autophagy, and hypermethylated and lowly expressed genes (Hyper-LGs) in estrogen receptor pathway and Wnt/β-catenin signaling pathway. Estrogen receptor 1 (*ESR1*), Erythrocyte membrane protein band 4.1 like 3 *(EPB41L3)*, Endothelin receptor B *(EDNRB)*, Inhibitor of DNA binding 4 *(ID4)* and placenta-specific 8 (*PLAC8*) were hub genes. Kaplan–Meier method was used to evaluate survival data of each identified gene. Lower expression levels of *ESR1* and *EPB41L3* were correlated with a shorter survival time. GSEA results showed that ‘cell adhesion molecules’ was the most enriched item. This research inferred the candidate genes and pathways that might be used in the diagnosis, treatment, and prognosis of cervical cancer.

## Introduction

Cervical cancer is the most prevalent reproductive malignancy, with an estimated 569847 new cases and 311365 deaths worldwide in 2018. Although its prognosis has been improved by the combination of screening and surgery, the disease still brings the fourth highest morbidity and mortality among females worldwide [4].

Human papillomavirus (HPV) is one player in cervical cancer, however, not all the infected patients develop the cancer ultimately, indicating that HPV infection is a necessary but not sufficient pathogenic condition [42]. Additionally, genetic and epigenetic alternations may also be involved [30,40].

Epigenetics focuses on heritable change in gene expression not mediated by changes within DNA sequence [3]. Altered methylation in DNA bases, either hypomethylation or hypermethylation, is a key event in carcinogenesis [17]. As cervical cancer progresses, epigenetic alteration rises to a level high enough to change gene expression. Epigenetic regulatory mechanisms in cervical cancer include DNA methylation, the hottest topic in this area, and post-translational modifications of histone proteins [10].

Based on high-throughput platforms, microarrays have emerged to screen genetic or epigenetic alternations in cancers in recent years [20]. Some differentially expressed genes (DEGs) in cervical intraepithelial neoplasia have been identified with this tool [46]. In addition, to explore differentially methylated genes (DMGs), aberrant methylation analysis has been applied [43]. However, these studies did not carry out a conjoint analysis that may result in insufficient power to discover key genes and pathways related to multiple cellular process and biological function. Differentially expressed and methylated genes can be detected using gene expression and methylation microarray data.

In our study, data from gene expression microarrays (GSE9750), gene methylation microarrays (GSE46306), as well as the expression profiles of oncogenes and tumor suppressor genes (TSGs) were integrated and analyzed by a series of bioinformatics tools. We used R software to obtain DEGs and DMGs and Venn diagram to overlap three datasets. Analyses also included gene-enrichment analysis (GO) and pathways (KEGG), protein–protein interaction (PPI) network analysis, and the identification and validation of oncogenes and TSGs. In our study, we aimed to screen out the aberrantly methylated and differentially expressed genes and associated pathways in cervical carcinogenesis. Some oncogenes or TSGs might be applied as biomarkers or therapeutic targets for cervical cancer.

## Methods

### Microarray data profile

We searched the gene expression profile dataset (GSE9750) and methylation profile dataset (GSE46306) on Gene Expression Omnibus (GEO) database (https://www.ncbi.nlm.nih.gov/geo/). Based on the GPL570 platform [HG-U133_Plus_2] Affymetrix Human Genome U133 Plus 2.0 Array [12], data from GSE9750 covered 33 samples of cervical cancer and 24 normal cervix samples. Based on the GPL13534 ((Illumina HumanMethylation450 BeadChip (HumanMethylation450_15017482)) [38], the methylation-profile microarray data from GSE46306 covered 6 cervical cancer samples and 20 normal samples.

### Identification of DEGs and DMGs

To explore the DEGs and DMGs, we selected the ‘limma’ packages [29] to process in GSE9750 and GSE46306 datasets. For DEGs, cut-off criteria were set as |logFC| > 1, and the adj.*p*-value < 0.05. For DMGs, cut-off criteria were set as FDR < 0.05 and |logFC| > 0.2. Subsequently, oncogene and TSG lists were produced from two online databases (http://ongene.bioinfo-minzhao.org/; https://bioinfo.uth.edu/TSGene/index.html) respectively. Furthermore, we employed an online Venn diagram [7] (http://bioinfogp.cnb.csic.es/tools/venny/) to identify the intersection of nodes among DEGs, DMGs, oncogenes, and TSGs. As a result, up-regulated hypomethylated oncogenes as well as down-regulated hypermethylated TSGs were retrieved.

### PPI network construction

Based on the aforementioned oncogenes and TSGs, we built a PPI network with the Search Tool for the Retrieval of Interacting Genes (STRING) database [35] (https://string-db.org/cgi/input.pl).

### Pathway analysis of aberrantly methylated DEGs

Gene ontology (GO) function and Kyoto Encyclopedia of Genes and Genomes (KEGG) analyses were conducted for our selected genes with an online tool FunRich [26] (http://www.funrich.org/). *P*<0.05 was considered as statistically significant.

### Validation of the selected genes

To further validate the expression profiles of oncogenes and TSGs, online software Gene Expression Profiling Interactive Analysis (GEPIA) [36] (http://gepia.cancer-pku.cn/) was used. For validation of the aberrantly methylated differentiated expressed genes, Illumina Human Methylation 450 platform in Cancer Genome Atlas (TCGA) database was assessed. To validate the selected oncogenes/TSGs on the translational level, immunohistochemistry staining results of both normal and cancerous cervical samples were assessed using the Human Protein Atlas database (https://www.proteinatlas.org/). ROC curve analysis was conducted to distinguish the normal from the cancerous tissues. We used cBioPortal tool (cBio Cancer Genomics Portal, http://www.cbioportal.org/) to find out genetic alterations in the hub genes and correlation between messenger RNA (mRNA) expression and DNA methylation in cervical cancer.

### Gene Set Enrichment Analysis

In TCGA, 306 cervical cancer samples were divided into two groups in the light of the median expression level. In order to identify the five hub genes’ underlying functions and the enrichment of biological processes (BPs) in the ranked DEGs between the two groups, Gene Set Enrichment Analysis (GSEA) (http://software.broadinstitute.org/gsea/index.jsp) was performed [33]. Collection of annotated gene set of c2.cp.kegg.v6.0.symbols.gmt in Molecular Signatures Database (MSigDB, http://software.broadinstitute.org/gsea/msigdb/index.jsp) was selected as the reference gene sets. Additionally, we used ‘Clusterprofiler’ package in R to handle the datasets, and ‘Enrichplot’ package to tease out the enriched pathways of the crucial genes. The adj.*p*<0.05 was set as the cut-off criterion.

## Results

### DEGs and DMGs in cervical cancer

**Figure 1:**
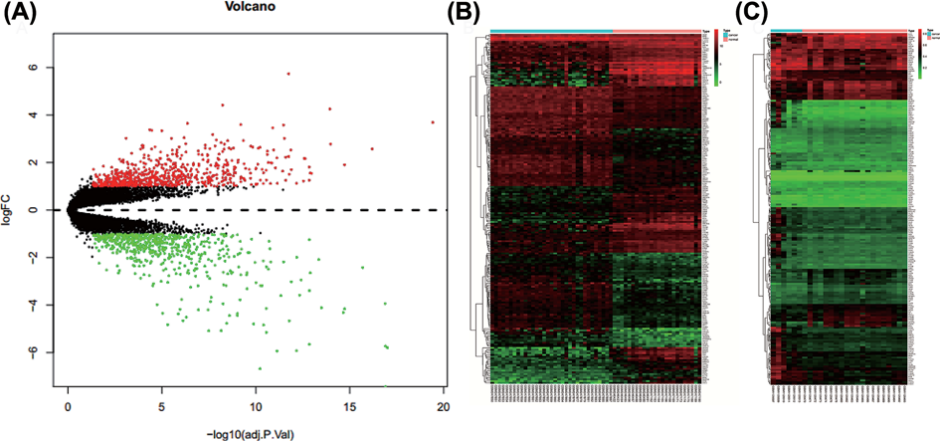
The results of DEGs in GSE9750, and DMGs in GSE46306 (**A**) Volcano Plot visualizing all the DEGs. Red dots represent up-regulated genes, green dots represent down-regulated genes, and black dots represent genes without differential expression. (**B**) Heat-map hierarchical clustering revealed the top 200 genes that were differentially expressed in cervical cancer (CC) groups when compared with control groups. Red and green colors indicate higher expression and lower expression, respectively. (**C**) Heat map of the DMPs. Hierarchical clustering showed separate groupings of DMPs for CC tissue and normal tissue.

The expression matrices from GSE9750 contained 33 cervical cancer samples and 24 normal samples. The DEGs were shown with a volcano plot and a clustering heat map (Figure 1A,B). We obtained 1313 DEGs, including 690 up-regulated genes and 623 down-regulated genes. A total of 1405 DMGs were obtained in GSE46306, as shown in the heat map (Figure 1C). As a result, 481 hypomethylated and 924 hypermethylated genes were also obtained.

### Aberrantly methylated DEGs

**Figure 2:**
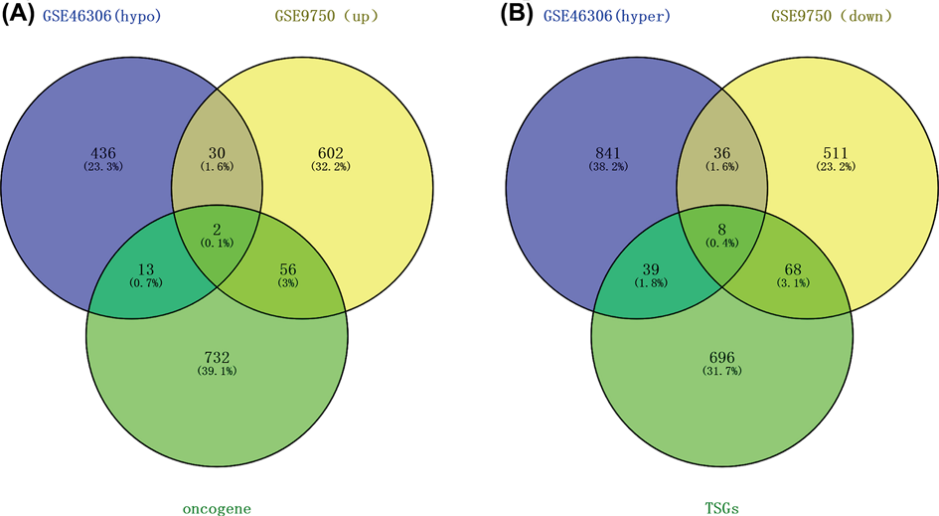
Aberrantly methylated and differentially expressed oncogenes and TSGs (**A**) Thirty-two hypomethylated and up-regulated genes were identified, including two oncogenes. (**B**) Forty-four hypermethylated and down-regulated genes were identified, including eight TSGs.

We got 32 hypomethylated and highly expressed genes (Hypo-HGs) and 44 Hyper-LGs. To pinpoint the aberrantly methylated DEGs, Hypo-HGs were overlapped with oncogenes, and Hyper-LGs with TSGs. Correspondingly, we identified two up-regulated hypomethylated oncogenes (Figure 2A), which may trigger tumorigenesis potentially by up-regulating gene expression after hypomethylation. Meanwhile, we retrieved eight down-regulated hypermethylated TSG (Figure 2B), indicating aberrant hypermethylation may trigger tumorigenesis through lowering the expression of these genes.

### PPI network construction

STRING online database was employed to construct PPI networks. Based on the Hypo-HGs, a PPI network containing 31 nodes and 60 edges was constructed, with a PPI enrichment *P*-value of 5.77e-09 (Supplementary Figure S1A). Based on the Hyper-LGs, a PPI network containing 44 nodes and 69 edges was constructed, with a PPI enrichment *P*-value of 1.79e-07 (Supplementary Figure S1B). Those genes were used for further functional analyses.

### Pathway analysis of aberrantly methylated DEGs

**Figure 3:**
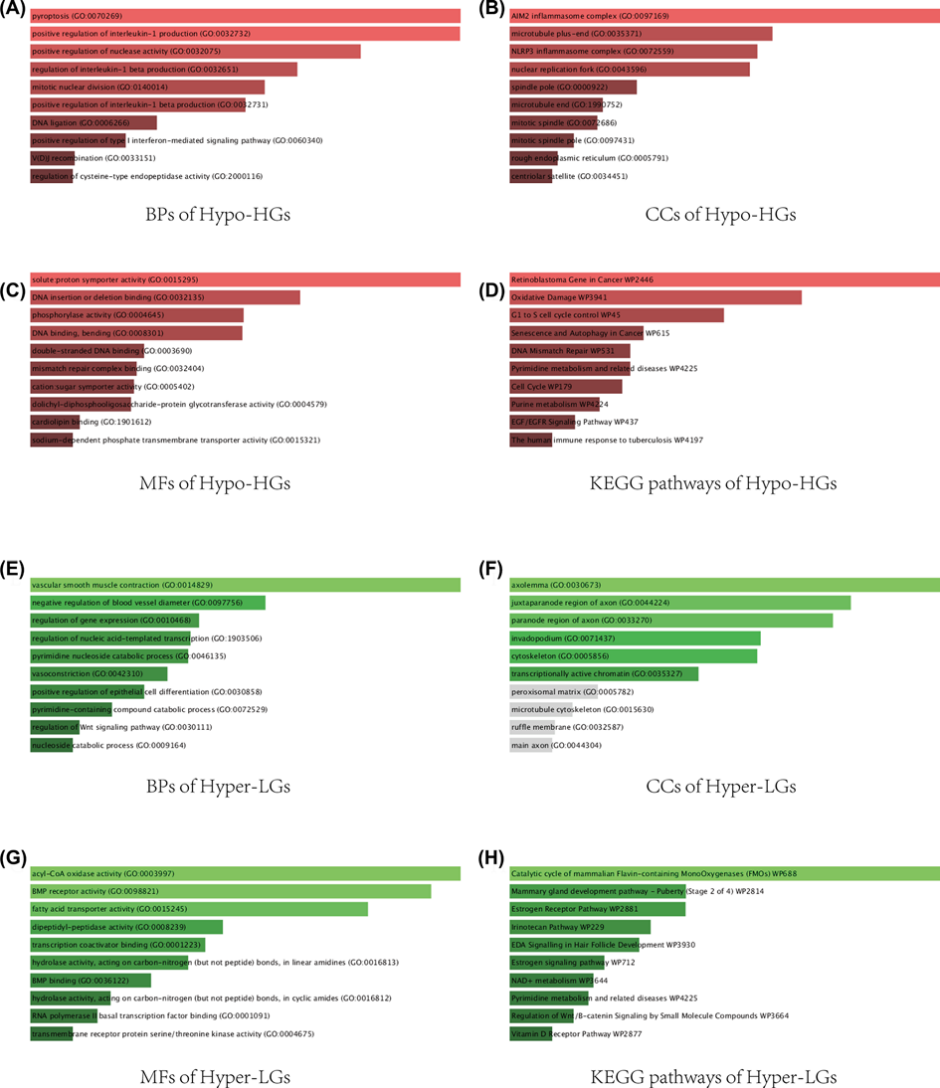
GO functional annotation and KEGG analysis of DEGs and DMGs (**A**) The top ten enriched BPs of Hypo-HGs (hypomethylated, highly expressed genes). (**B**) The top ten enriched CCs of Hypo-HGs. (**C**) The top ten enriched MFs of Hypo-HGs. (**D**) The top ten enriched KEGG pathways of Hypo-HGs. (**E**) The top ten enriched BPs of Hyper-LGs (hypermethylated, lowly expressed genes). (**F**) The top ten enriched CCs of Hyper-LGs. (**G**) The top ten enriched MFs of Hyper-LGs. (**H**) The top ten enriched KEGG pathways of Hyper-LGs.

There were biological process (BP), cellular component (CC), and molecular function (MF) in enriched GO terms. GO analysis showed that pyroptosis, positive regulation of interleukin-1 and positive regulation of nuclease activity for BP production were significantly enriched in Hypo-HGs (Figure 3A). For CC analysis, AIM2 inflammasome complex, microtubule plus-end and NLRP3 inflammasome complex were significantly enriched in Hypo-HGs (Figure 3B). For MF analysis, Hypo-HGs were significantly enriched in solute proton symporter activity, DNA insertion or deletion binding and phosphorylase activity (Figure 3C). KEGG analysis indicated that up-hypomethylated genes mainly contributed to cell cycle, senescence, and autophagy in cancer (Figure 3D).

In comparison, Hyper-LGs were mainly associated with (i) vascular smooth muscle contraction, negative regulation of blood vessel diameter, and regulation of gene expression (BP analysis, Figure 3E); (ii) axolemma, juxtaparanode region of axon, and paranode region of axon (CC analysis, Figure 3F); (iii) acly-CoA oxidase activity, BMP receptor activity, and fatty acid transporter activity (MF analysis, Figure 3G). These genes were mainly enriched in estrogen receptor pathway, estrogen signaling pathway and regulation of Wnt/β-catenin signaling by small molecules (KEGG analysis, Figure 3H).

### Validation of the selected genes

**Figure 4:**
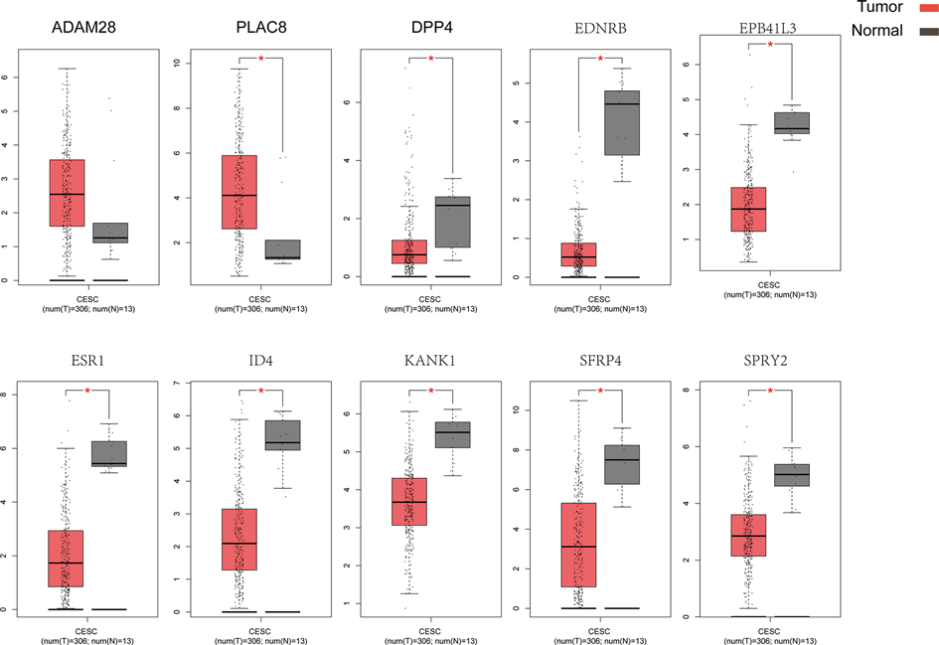
Validation of the ten genes in the GEPIA Box plots showing the ten genes in mRNA expression, using data from the TCGA database in GEPIA. The statuses of the expression of nine genes were the same as in our study. *：*P*-values<0.05. (*ADAM28* was not statistically significant).

**Figure 5:**
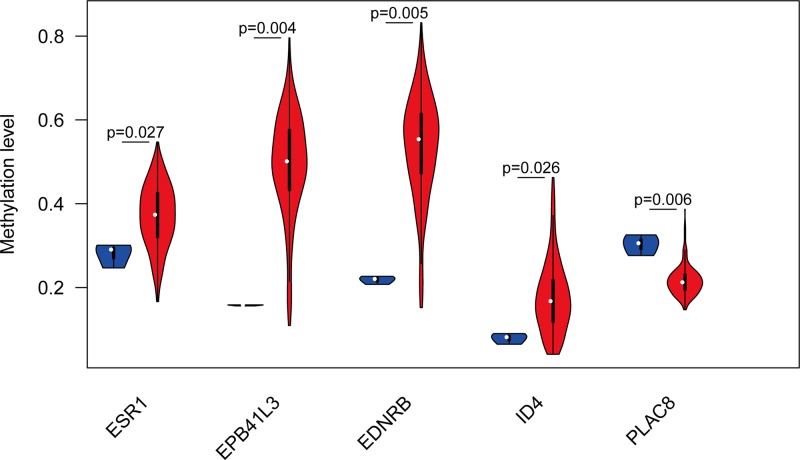
Validation of the ten genes in the TCGA database Violin plots showing the methylation status of the five genes was in accordance with the data from the TCGA database.

**Figure 6:**
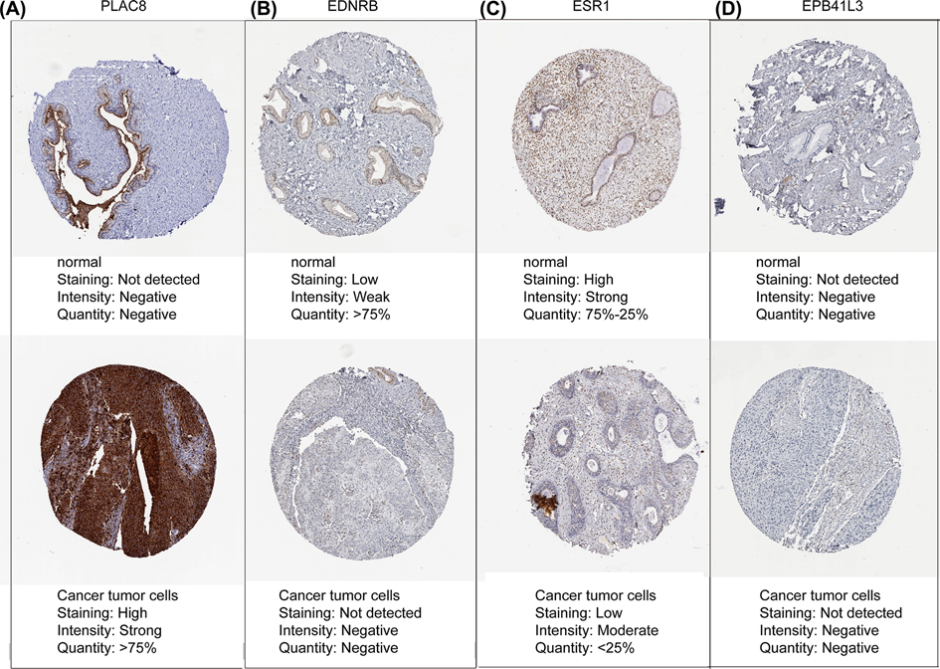
Immunohistochemistry of the four genes based on The Human Protein Atlas (**A**) Protein levels of PLAC8 in cancer tissues (staining: high; intensity: strong; quantity: >75%). Protein levels of PLAC8 in the normal tissues (staining: negative; intensity: negative; quantity: none). (**B**) Protein levels of ENDRB in cancer tissues (staining: negative; intensity: negative; quantity: none). Protein levels of ENDRB in the normal tissues (staining: low; intensity: weak; quantity: >75%). (**C**) Protein levels of ESR1 in cancer tissues (staining: low; intensity: moderate; quantity: <25%). Protein levels of ESR1 in the normal tissues (staining: high; intensity: strong; quantity: 75-25%). (**D**) Protein levels of EPB41L3 in the tumor tissues (staining: negative; intensity: negative; quantity: none). Protein levels of EPB41L3 in the normal tissues (staining: negative; intensity: negative; quantity: none).

**Figure 7:**
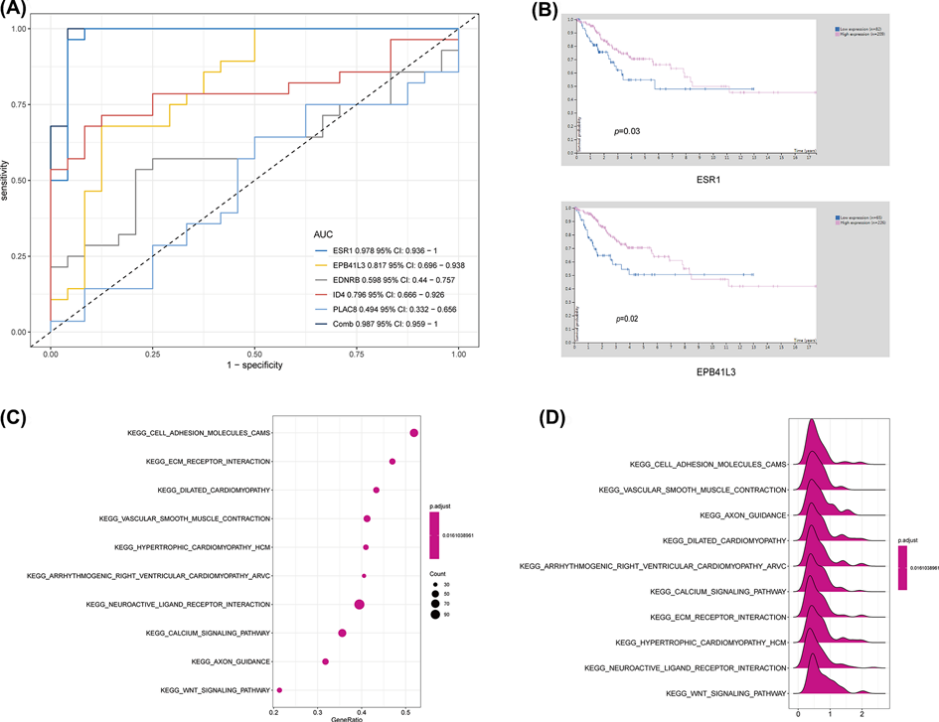
ROC curve analysis, suvival analysis and function analysis of the hub genes (**A**) ROC curve analysis was implemented to evaluate the capacity of five genes to distinguish the cervical cancer from the normal tissues. (**B**) The prognostic significance of hub genes in cervical cancer (CSCC). Lower levels of *ESR1* and *EPB41L3* were associated with poorer overall survival time, respectively. (**C,D**) GSEA using TCGA CESC databases. The ten most functional gene sets enriched in CESC samples.

We employed data from GEPIA to validate the role of the differentially expressed and methylated genes in carcinogenesis. Two upregulated-hypomethylated oncogenes, along with eight downregulated-hypermethylated TSGs, showed differential expression between normal tissues and cancer tissues (Figure 4). In addition, based on TCGA CESC data, we identified five DMGs: *ESR1, EPB41L3, EDNRB, ID4*, and *PLAC8* (Figure 5). Additionally, the immunohistochemistry staining images from Human Protein Atlas database indicated the up-regulation of *PLAC8* gene and the down-regulation of *ESR1* and *EDNRB* genes. Besides, the expression of *EPB41L3* showed no significant difference between normal tissues and tumor tissues, and immunohistochemistry staining of *ID4* was not obtained in Human Protein Atlas database (Figure 6). Moreover, *ESR1, EPB41L3*, and *ID4* all showed an AUC of more than 0.7 and the combined diagnostic power of the five genes was 0.987 (Figure 7A), which were enough to distinguish cervical cancer tissues from the normal tissues.

Furthermore, Human Protein Atlas database was applied to gain survival time and gene expression levels, to assess the prognostic significance of the five aberrantly methylated and differentially expressed genes. Kaplan–Meier method was used to evaluate survival time data of each identified gene. Analysis demonstrated lower expression levels of *ESR1* and *EPB41L3* were correlated with shorter survival time (Figure 7B).

### GSEA

To explore the functions of five genes in cervical cancer, we conducted GSEA to search pathways enriched in the TCGA samples. Ten enriched pathways in the gene sets (*n*=306) included cell adhesion molecules (CAMs), Wnt signaling pathway, axon guidance, calcium signaling pathway, neuroactive ligand receptor interaction, arrhythmogenic right ventricular cardiomyopathy ARVC, hypertrophic cardiomyopathy HCM, vascular smooth muscle contraction, dilated cardiomyopathy, and ECM receptor interaction (adj.*P*<0.05) (Figure 7C,D).

### Genetic information of the five genes

**Figure 8:**
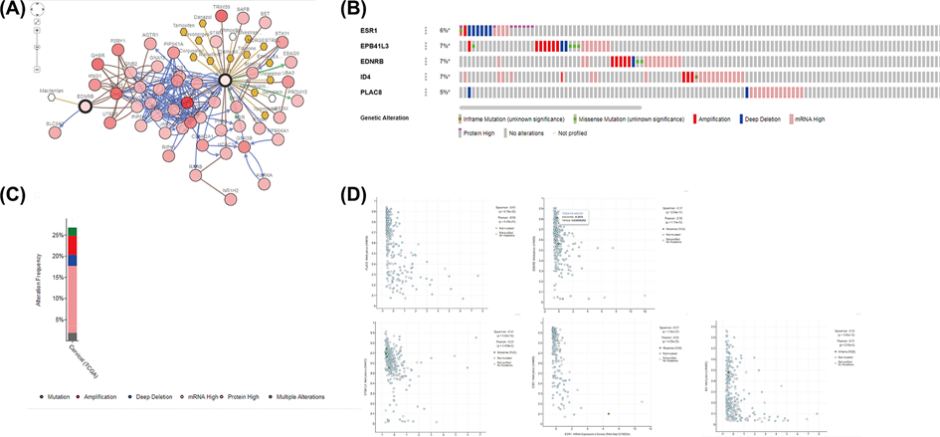
Genetic alterations in the five genes (**A**) The network contained 52 nodes, including two query genes and 50 most interactive neighbor genes. The relationship between hub genes and drugs. (**B**) A visual summary across a set of CESC (data from cervical squamous cell carcinoma TCGA, provisional) showed the genetic alteration of the seven genes appeared in 82 (27%) of the 308 sequenced cases/patients (310 total). (**C**) An overview of changes in the five genes in the genomics datasets of TCGA. (**D**) Correlation between mRNA and DNA methylation in the five genes in TCGA CESC patients.

The genetic alteration in the five genes was analyzed with cBioPortal software. The network constructed by *ESR1* and *EDNRB* and their 50 most associated neighbor genes were exhibited (only two of the five genes had a joint node, while the remaining three genes had no junctions and were not shown). In addition, drugs targeting the five genes were shown, only *ESR1* and *EDNRB* were taken for drug targets currently (Figure 8A). Figure 8B,C illustrated that the five genes were changed in 82 (27%) of the 308 cases/patients (310 in total); *EDNRB, EPB41L3*, and *ID4* showed most diverse alterations, including amplification, missense mutation etc. The relevance between mRNA and DNA methylation of the five genes in the TCGA CESC patients was demonstrated in Figure 8D. We found that methylation negatively regulated the mRNA expression of the five genes.

## Dicussion

The development of cervical cancer was a complicated process. Elucidating the underlying molecular mechanisms of oncogenesis would extremely benefit the diagnosis, treatment and prognosis evaluation of cervical cancer. Database-based bioinformatics analysis is increasingly adopted to screen out targeted biological molecules that might have a guiding role in the diagnosis and treatment of tumors [20].

In the present study, we utilized NCBI-GEO to analyze the data of gene expression (GSE9750) and gene methylation (GSE46306) microarrays for cervical cancer. We used the R software to screen genes which were differentially expressed.

In our research, we identified two up-regulated hypomethylated oncogenes and eight down-regulated hypermethylated TSGs in cervical cancer. To predict the protein interaction among these genes, PPI network was constructed using the online STRING database. Furthermore, based on TCGA CESC data, we obtained five DMGs. Functional and enrichment analyses demonstrated the pathways and hub genes regulated by aberrant methylation. Meanwhile, the Kaplan–Meier analysis illustrated that lower levels of *EPB41L3* and *ESR1* were relevant to shorter survival time of CESC patients.

As was implied by FunRich analysis, Hypo-HGs were significantly associated with cell cycle and autophagy. Increasing evidence indicates that aberrant cell cycle plays a crucial role in the development of cancer [45], due to the dysregulation of TSGs or oncogenes, such as *p53, p16*, and cyclin D1, etc. [31]. Cyclin D1 is essential for cell cycle G_1_ [2]. Cyclin D1 aberrantly expressed is implicated in the progression of cervical cancer [1]. Cell cycle could participate significantly in the tumorigenesis of cervical cancer due to cell cycle regulators. Studies have explored the dual effects of autophagy in cancer. How autophagy functions in tumor suppression has been explained, like cell death, senescence, and metabolic stress [18]. Autophagy facilitates neoplasm growth by supporting cellular metabolism. Inhibiting autophagy has become a rationale in cancer therapy development [19].

For Hyper-LGs in cervical cancer, estrogen receptor pathway and Wnt/β-catenin signaling pathway were enriched. Multi-parity and oral contraceptives usages, particular long-term usage, are powerful risks of cervical cancer in HPV-infected women, raising the potential that estrogen and HPV infection might exert synergistic effect to cervical cancer development [28]. Previous studies have discovered that loss of exogenous estrogen, cervical disease progressed slowly and pre-existing neoplasia regressed partially in HPV transgenic mice. In addition, treated with estrogen, ERα-deficient HPV transgenic mice did not show faster development of cervical cancer [8]. Therefore, estrogen and ERα might play a key part in the tumorigenesis of cervical cancer. The Wnt/β-catenin signaling pathway, through activating β-catenin to initiate its downstream target genes transcription, could promote tumorigenesis, invasion, and metastasis [44]. Previous study found that deprivation of Wnt2 inhibited SiHa cell migration and invasion, as well as reversed EMT by restraining theWnt2/β-catenin pathway. Moreover, overexpression of Wnt2 led to metastasis in cervical cancer, which was associated with activation of β-catenin and induction of EMT [48]. In conclusion, Wnt/β-catenin signaling pathway aberrantly activated could enhance cervical cancer metastasis and invasion.

GEPIA was employed to validate the role of the ten selected genes in carcinogenesis. Two up-regulated hypomethylated oncogenes and eight down-regulated hypermethylated TSGs showed expression difference between cancer samples and normal samples. We next accessed TCGA CESC data to validate the performance of five DMGs, including *ESR1, EPB41L3, EDNRB, ID4*, and *PLAC8*. The results of boxplots based on TCGA database were mostly consistent with the data of GEO analysis. We further used immunohistochemistry staining to verify the deregulation of the gene expression. Last, we performed ROC curve analysis to assess the capacity of five genes to distinguish cervical cancer from the normal tissues.

These five hub genes are involved in tumor progression. *PLAC8* is a cysteine-rich protein highly expressed in giant trophoblasts and spongiotrophoblast layer in placenta [11]. In a recent study, *PLAC8* could facilitate the EMT in nasopharyngeal carcinoma [15]. In addition, high expression of *PLAC8* in clear cell renal carcinoma was correlated to malignant progression and poor prognosis. [32]. In our study, we found that the expression of *PLAC8* was increased by hypomethylation, demonstrating that *PLAC8* may function as an initiator of cervical cancer proliferation.

*EPB41L3* is a TSG which facilitates tumor cell apoptosis and inhibits its proliferation [21]. Overexpressed of *EPB41L3* could enhance the migration and invasion of multiple cancers, such as esophageal cancer [47], lung cancer [22], and hepatocellular carcinoma [49]. Methylation of *EPB41L3* was lower than or equal to that in CIN1 group, but rose significantly till to the peak in CIN2/3 group [41]. Our study showed that lower expression levels of *EPB41L3* was related to shorter survival time. As a tumor suppressor, *ID4* could promote the proliferation and inhibit the apoptosis of tumor cells in prostate [23], lung [5], and gastric [6] cancers. Expression of *ID4* is lowered by its promoter hypermethylation [25]. In the present study, *EPB41L3* and *ID4* had a possibility of 7% mutation in cervical cancer, so we speculated that the mutation led to the aberrant methylation or deregulation of both genes. Furthermore, hypermethylation of *EPB41L3* and *ID4* may suppress the development of cervical cancer.

*ESR1*, a nuclear hormone receptor and oncoprotein, is expressed in approximately 70% of breast cancers [34]. *ESR1* may serve to develop a hormonal therapy for breast cancer, since *ESR1* gene mutating is related to acquired endocrine resistance in patients with ER-positive metastatic breast cancer [27]. In the present study, lower expression level of *ESR1* was associated with shorter survival time. Previous research reported that *EDNRB* was methylated in prostate adenocarcinoma [24]. The methylation of *EDNRB* gene promoter was associated with gastric cancer invasion [37]. In our study, we found that *ESR1* and *EDNRB* expression levels were lower in cervical cancer samples than in the normal samples. Aberrant methylation may contribute to these differences. The rate of *ESR1* and *EDNRB* mutation was 6 and 7% in cervical cancer. Therefore, we speculated that the mutation resulted in the aberrant methylation or down-regulation of *ESR1* and *EDNRB*. Our study implied that *ESR1* and *EDNRB* may serve as targets in the creation of anti-tumor drugs.

GSEA was conducted to identify the functions of the five genes involved in cervical cancer. CAMs were enriched in TCGA samples. CAMs participate in various BPs, like differentiation, growth and apoptosis, and facilitate cellular interaction and migration [9,13,14]. In highly aggressive tumors, CAMs might show decreased immunoexpression [16]. Cervical cancers and their precursor lesions demonstrated the absence or alteration in cellular adherence [39]. During the development of cervical lesions, CAMs expression is altered either in location (cytoplasmic or membrane localization) or in quantity. E-cadherin is a number of CAM family that is aberrantly expressed in cervical lesions and leads to the invasion and metastasis of this neoplasm [9]. Taken together, low expression of CAMs could facilitate the metastasis of cervical cancer.

In conclusion, we conducted integrated bioinformatic analysis to identify aberrantly methylated and differentially expressed oncogenes and TSGs in cervical cancer tissues, as well as the related pathways and functions. Ten genes validated with the TCGA database, including five DMGs (*ESR1, EPB41L3, EDNRB,ID4, PLAC*), might be used as biomarkers and therapeutic targets in the precise diagnosis and treatment of cervical cancer.

The limitations in our study are as follows. First, since our research is a data analysis, biological experiments are in urgent need to validate our results. Second, it is necessary to explore the molecular mechanism in detail. In the future, we will deepen our research with well-designed experiments (including PCR, Western blot, BSP and COBRA).

## Supplementary Material

Supplementary Figure S1

## Data Availability

The datasets used or analyzed during the current study are available from the GEO (https://www.ncbi.nlm.nih.gov) and Cancer Genome Atlas database (https://cancergenome.nih.gov).

## Abbreviations

AIM2: absent in melanoma 2
BMP: bone morphogenetic protein
BP: biological process
BSP: bisulfite sequencing PCR
CAM: cell adhesion molecule
CC: cellular component
CESC: cervical squamous cell carcinoma
CIN: cervical Intraepithelial neoplasia
COBRA: combined bisulfite restriction analysis
DEG: differentially expressed gene
DMG: differentially methylated gene
EDNRB: endothelin receptor B
EMT: epithelial-mesenchymal transition
EPB41L3: erythrocyte membrane protein band 4.1 like 3
ERα: estrogen receptor α
ESR1: estrogen receptor 1
GEO: gene expression omnibus
GEPIA: gene expression profiling interactive analysis
GSEA: gene set enrichment analysis
HPV: human papillomavirus
Hypo-HG: hypomethylated and highly expressed gene
ID4: inhibitor of DNA binding 4
MF: molecular function
MSigDB: molecular signatures database
mRNA: messenger RNA
NLRP3: NLR family pyrin domain containing 3
PLAC8: placenta-specific 8
PPI: protein–protein interaction
ROC: receiver operating characteristic
STRING: search tool for the retrieval of interacting genes
TSG: tumor suppressor gene

## References

[1] Bae D.S., Cho S.B., Kim Y.J., Whang J.D., Song S.Y., Park C.S. et al. (2001) Aberrant expression of cyclin D1 is associated with poor prognosis in early stage cervical cancer of the uterus. Gynecol. Oncol. 81, 341–347 10.1006/gyno.2001.6196 11371120

[2] Baldin V., Lukas J., Marcote M.J., Pagano M. and Draetta G. (1993) Cyclin D1 is a nuclear protein required for cell cycle progression in G1. Genes Dev. 7, 812–821 8491378 10.1101/gad.7.5.812

[3] Bird A. (2002) DNA methylation patterns and epigenetic memory. Genes Dev. 16, 6–21 11782440 10.1101/gad.947102

[4] Bray F., Ferlay J., Soerjomataram I., Siegel R.L., Torre L.A. and Jemal A. (2018) Global cancer statistics 2018: GLOBOCAN estimates of incidence and mortality worldwide for 36 cancers in 185 countries. CA Cancer J. Clin. 68, 394–424 30207593 10.3322/caac.21492

[5] Castro M., Grau L., Puerta P., Gimenez L., Venditti J., Quadrelli S. et al. (2010) Multiplexed methylation profiles of tumor suppressor genes and clinical outcome in lung cancer. J. Transl. Med. 8, 86 10.1186/1479-5876-8-86 20849603 PMC2955578

[6] Chan A.S., Tsui W.Y., Chen X., Chu K.M., Chan T.L., Chan A.S. et al. (2003) Downregulation of ID4 by promoter hypermethylation in gastric adenocarcinoma. Oncogene 22, 6946–6953 10.1038/sj.onc.1206799 14534543

[7] Chen H. and Boutros P.C. (2011) VennDiagram: a package for the generation of highly-customizable Venn and Euler diagrams in R. BMC Bioinformatics 12, 35 10.1186/1471-2105-12-35 21269502 PMC3041657

[8] Chung S.H., Franceschi S. and Lambert P.F. (2010) Estrogen and ERalpha: culprits in cervical cancer? Trends Endocrinol. Metab. 21, 504–511 10.1016/j.tem.2010.03.005 20456973 PMC2914219

[9] de Mendez M.T. and Bosch A.L. (2011) Abnormal immunoexpression of cell adhesion molecules (CAMs) in cervical cancer. Int. J. Surg. Pathol. 19, 733–742 10.1177/1066896909343435 20643667

[10] Fang J., Zhang H. and Jin S. (2014) Epigenetics and cervical cancer: from pathogenesis to therapy. Tumour Biol. 35, 5083–5093 10.1007/s13277-014-1737-z 24554414

[11] Galaviz-Hernandez C., Stagg C., de Ridder G., Tanaka T.S., Ko M.S., Schlessinger D. et al. (2003) Plac8 and Plac9, novel placental-enriched genes identified through microarray analysis. Gene 309, 81–89 10.1016/S0378-1119(03)00508-0 12758124

[12] Gautier L., Cope L., Bolstad B.M. and Irizarry R.A. (2004) affy–analysis of Affymetrix GeneChip data at the probe level. Bioinformatics 20, 307–315 10.1093/bioinformatics/btg405 14960456

[13] Haga T., Uchide N., Tugizov S. and Palefsky J.M. (2008) Role of E-cadherin in the induction of apoptosis of HPV16-positive CaSki cervical cancer cells during multicellular tumor spheroid formation. Apoptosis 13, 97–108 10.1007/s10495-007-0132-2 17906929

[14] Hirohashi S. (1998) Inactivation of the E-cadherin-mediated cell adhesion system in human cancers. Am. J. Pathol. 153, 333–339 10.1016/S0002-9440(10)65575-7 9708792 PMC1852964

[15] Huang M.L., Zou Y., Yang R., Jiang Y., Sheng J.F., Han J.B. et al. (2019) Placenta specific 8 gene induces epithelial-mesenchymal transition of nasopharyngeal carcinoma cells via the TGF-beta/Smad pathway. Exp. Cell Res. 374, 172–180 10.1016/j.yexcr.2018.11.021 30496758

[16] Ivanov D.B., Philippova M.P. and Tkachuk V.A. (2001) Structure and functions of classical cadherins. Biochem. Biokhim. 66, 1174–1186 10.1023/A:1012445316415 11736639

[17] Jablonska E. and Reszka E. (2017) Selenium and epigenetics in cancer: focus on DNA methylation. Adv. Cancer. Res. 136, 193–234 10.1016/bs.acr.2017.07.002 29054419

[18] Jin S. and White E. (2007) Role of autophagy in cancer: management of metabolic stress. Autophagy 3, 28–31 10.4161/auto.3269 16969128 PMC2770734

[19] Kimmelman A.C. and White E. (2017) Autophagy and tumor metabolism. Cell Metab. 25, 1037–1043 10.1016/j.cmet.2017.04.004 28467923 PMC5604466

[20] Kulasingam V. and Diamandis E.P. (2008) Strategies for discovering novel cancer biomarkers through utilization of emerging technologies. Nat. Clin. Pract. Oncol. 5, 588–599 10.1038/ncponc1187 18695711

[21] Li X., Zhang Y., Zhang H., Liu X., Gong T., Li M. et al. (2011) miRNA-223 promotes gastric cancer invasion and metastasis by targeting tumor suppressor EPB41L3. Mol. Cancer Res. 9, 824–833 10.1158/1541-7786.MCR-10-0529 21628394

[22] Liang H., Yan X., Pan Y., Wang Y., Wang N., Li L. et al. (2015) MicroRNA-223 delivered by platelet-derived microvesicles promotes lung cancer cell invasion via targeting tumor suppressor EPB41L3. Mol. Cancer 14, 58 10.1186/s12943-015-0327-z 25881295 PMC4360939

[23] Nasif D., Campoy E., Laurito S., Branham R., Urrutia G., Roque M. et al. (2018) Epigenetic regulation of ID4 in breast cancer: tumor suppressor or oncogene? Clin. Epigenetics 10, 111 10.1186/s13148-018-0542-8 30139383 PMC6108146

[24] Nelson J.B., Lee W.H., Nguyen S.H., Jarrard D.F., Brooks J.D., Magnuson S.R. et al. (1997) Methylation of the 5′ CpG island of the endothelin B receptor gene is common in human prostate cancer. Cancer Res. 57, 35–37 8988036

[25] Patel D., Morton D.J., Carey J., Havrda M.C. and Chaudhary J. (2015) Inhibitor of differentiation 4 (ID4): from development to cancer. Biochim. Biophys. Acta 1855, 92–103 25512197 10.1016/j.bbcan.2014.12.002 PMC4312723

[26] Pathan M., Keerthikumar S., Ang C.S., Gangoda L., Quek C.Y., Williamson N.A. et al. (2015) FunRich: an open access standalone functional enrichment and interaction network analysis tool. Proteomics 15, 2597–2601 10.1002/pmic.201400515 25921073

[27] Pritchard K.I. (2013) Endocrine therapy: is the first generation of targeted drugs the last? J. Intern. Med. 274, 144–152 10.1111/joim.12065 23844917

[28] Ramachandran B. (2017) Functional association of oestrogen receptors with HPV infection in cervical carcinogenesis. Endocr. Relat. Cancer 24, R99–R108 10.1530/ERC-16-0571 28283546

[29] Ritchie M.E., Phipson B., Wu D., Hu Y., Law C.W., Shi W. et al. (2015) limma powers differential expression analyses for RNA-sequencing and microarray studies. Nucleic Acids Res. 43, e47 10.1093/nar/gkv007 25605792 PMC4402510

[30] Rogeri C.D., Silveira H.C.S., Causin R.L., Villa L.L., Stein M.D., de Carvalho A.C. et al. (2018) Methylation of the hsa-miR-124, SOX1, TERT, and LMX1A genes as biomarkers for precursor lesions in cervical cancer. Gynecol. Oncol. 150, 545–551 10.1016/j.ygyno.2018.06.014 29960712

[31] Schafer K.A. (1998) The cell cycle: a review. Vet. Pathol. 35, 461–478 10.1177/030098589803500601 9823588

[32] Shi L., Xiao L., Heng B., Mo S., Chen W. and Su Z. (2017) Overexpression of placenta specific 8 is associated with malignant progression and poor prognosis of clear cell renal cell carcinoma. Int. Urol. Nephrol. 49, 1165–1176 10.1007/s11255-017-1578-y 28349447

[33] Subramanian A., Kuehn H., Gould J., Tamayo P. and Mesirov J.P. (2007) GSEA-P: a desktop application for Gene Set Enrichment Analysis. Bioinformatics 23, 3251–3253 10.1093/bioinformatics/btm369 17644558

[34] Swetzig W.M., Wang J. and Das G.M. (2016) Estrogen receptor alpha (ERalpha/ESR1) mediates the p53-independent overexpression of MDM4/MDMX and MDM2 in human breast cancer. Oncotarget 7, 16049–16069 10.18632/oncotarget.7533 26909605 PMC4941297

[35] Szklarczyk D., Franceschini A., Wyder S., Forslund K., Heller D., Huerta-Cepas J. et al. (2015) STRING v10: protein-protein interaction networks, integrated over the tree of life. Nucleic Acids Res. 43, D447–D452 10.1093/nar/gku1003 25352553 PMC4383874

[36] Tang Z., Li C., Kang B., Gao G., Li C. and Zhang Z. (2017) GEPIA: a web server for cancer and normal gene expression profiling and interactive analyses. Nucleic Acids Res. 45, W98–W102 10.1093/nar/gkx247 28407145 PMC5570223

[37] Tao K., Wu C., Wu K., Li W., Han G., Shuai X. et al. (2012) Quantitative analysis of promoter methylation of the EDNRB gene in gastric cancer. Med. Oncol. 29, 107–112 10.1007/s12032-010-9805-8 21264540

[38] Tian Y., Morris T.J., Webster A.P., Yang Z., Beck S., Feber A. et al. (2017) ChAMP: updated methylation analysis pipeline for Illumina BeadChips. Bioinformatics 33, 3982–3984 10.1093/bioinformatics/btx513 28961746 PMC5860089

[39] Van de Putte G., Kristensen G.B., Baekelandt M., Lie A.K. and Holm R. (2004) E-cadherin and catenins in early squamous cervical carcinoma. Gynecol. Oncol. 94, 521–527 10.1016/j.ygyno.2004.05.046 15297198

[40] van Leeuwen R.W., Ostrbenk A., Poljak M., van der Zee A.G.J., Schuuring E. and Wisman G.B.A. (2019) DNA methylation markers as a triage test for identification of cervical lesions in a high risk human papillomavirus positive screening cohort. Int. J. Cancer 144, 746–754 10.1002/ijc.31897 30259973 PMC6587981

[41] Vasiljevic N., Scibior-Bentkowska D., Brentnall A.R., Cuzick J. and Lorincz A.T. (2014) Credentialing of DNA methylation assays for human genes as diagnostic biomarkers of cervical intraepithelial neoplasia in high-risk HPV positive women. Gynecol. Oncol. 132, 709–714 10.1016/j.ygyno.2014.02.001 24508839 PMC3989115

[42] Walboomers J.M., Jacobs M.V., Manos M.M., Bosch F.X., Kummer J.A., Shah K.V. et al. (1999) Human papillomavirus is a necessary cause of invasive cervical cancer worldwide. J. Pathol. 189, 12–19 10.1002/(SICI)1096-9896(199909)189:1<12::AID-PATH431>3.0.CO;2-F 10451482

[43] Wang C.H., Li Y.H., Tian H.L., Bao X.X. and Wang Z.M. (2018) Long non-coding RNA BLACAT1 promotes cell proliferation, migration and invasion in cervical cancer through activation of Wnt/beta-catenin signaling pathway. Eur. Rev. Med. Pharmacol. Sci. 22, 3002–3009 29863244 10.26355/eurrev_201805_15057

[44] Wang X.B., Cui N.H., Liu X.N., Ma J.F., Zhu Q.H., Guo S.R. et al. (2018) Identification of DAPK1 promoter hypermethylation as a biomarker for intra-epithelial lesion and cervical cancer: a meta-analysis of published studies, TCGA, and GEO Datasets. Front. Genet. 9, 258 30065752 10.3389/fgene.2018.00258 PMC6056635

[45] Wiman K.G. and Zhivotovsky B. (2017) Understanding cell cycle and cell death regulation provides novel weapons against human diseases. J. Intern. Med. 281, 483–495 10.1111/joim.12609 28374555

[46] Yao S. and Liu T. (2018) Analysis of differential gene expression caused by cervical intraepithelial neoplasia based on GEO database. Oncol. Lett. 15, 8319–8324 29805564 10.3892/ol.2018.8403 PMC5950031

[47] Zeng R., Liu Y., Jiang Z.J., Huang J.P., Wang Y., Li X.F. et al. (2018) EPB41L3 is a potential tumor suppressor gene and prognostic indicator in esophageal squamous cell carcinoma. Int. J. Oncol. 52, 1443–1454 29568917 10.3892/ijo.2018.4316 PMC5873871

[48] Zhou Y., Huang Y., Cao X., Xu J., Zhang L., Wang J. et al. (2016) WNT2 promotes cervical carcinoma metastasis and induction of epithelial-mesenchymal transition. PLoS ONE 11, e0160414 10.1371/journal.pone.0160414 27513465 PMC4981407

[49] Zhu L., Yang N., Chen J., Zeng T., Yan S., Liu Y. et al. (2017) LINC00052 upregulates EPB41L3 to inhibit migration and invasion of hepatocellular carcinoma by binding miR-452-5p. Oncotarget 8, 63724–63737 28969024 10.18632/oncotarget.18892 PMC5609956

