# Supplementary material for: Identification of crucial aberrantly methylated and differentially expressed genes related to cervical cancer using an integrated bioinformatics analysis
Source: Biosci Rep. 2020 May 12;40(5):BSR20194365. doi: 10.1042/BSR20194365 (PMC7218222; doi:10.1042/BSR20194365)
Supplement: Supplementary Figure S1 [file BSR-2019-4365_supp.pdf]

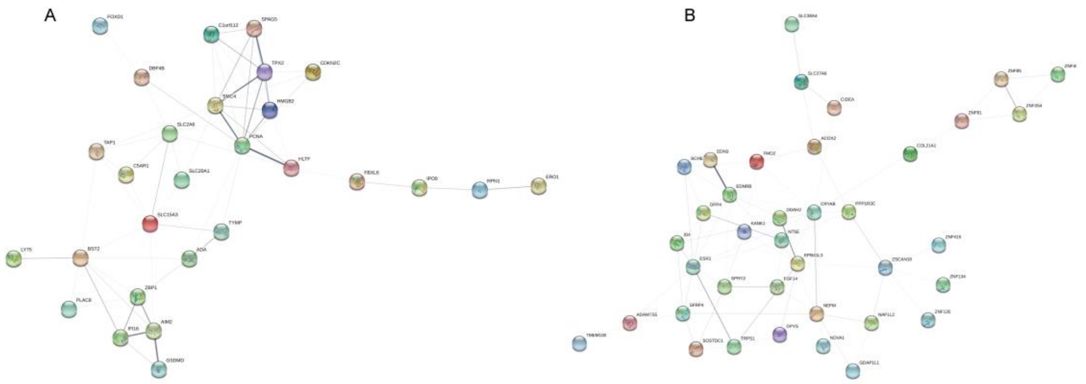

Figure S1 PPI network for the DEGs and DMGs. (A) A total of 27 genes were filtered into the upregulated and hypomethylated PPI network that contained 44 nodes and 69 edges. (B) A total of 45 genes were mapped into the downregulated and hypermethylated PPI network that contained 31 nodes and 60 edges.
